# Microscopy of the Teeth

**Published:** 1868-11

**Authors:** S. P. Cutler

**Affiliations:** Recently Professor of Chemistry and Microscopy in the Ohio Dental College, Cincinnati. Formerly Professor of Chemistry and Natural Sciences in the Botanic Medical College, Memphis, Tenn.


### MICROSCOPY OF THE TEETH.

BY S. P. CUTLER, M. D., A. E. G., D. D. S.

Recently Professor of Chemistry and Microscopy in the Ohio Dental College, Cincinnati. Formerly Professor of Chemistry and Natural Sciences in the Botanic Medical College, Memphis, Tenn.

I notice in the proceedings of the Niagara Association that exceptions were taken to my publications concerning the dentinal nerve fibrils, because I had not given my processes of preparing specimens so that others could follow my researches, as is customary in making new scientific announcements, so that they might be enabled to arrive at similar conclusions. This is all right. The profession have an undoubted right to all of my labors, and shall have them in detail, leaving out my many abortive attempts before arriving at satisfactory conclusions. One reason why I have not given them sooner is that my researches were not, nor are they yet fully perfected. Neither did I expect the profession to acknowledge any of my discoveries at once, this is not generally the case in such intricate and recondite subjects which require a thorough knowledge of the use of the microscope as well as a thorough knowledge of the subject treated of, namely, histology in its highest attributes, which I am glad to know many in the profession are aiming at. This is just what I wish to see, and if I have in any way contributed to this end, I am amply rewarded even if all of my own researches and conclusions should stand for ever unacknowledged.

In giving my processes I would like very much for all who have instruments to follow me and give their individual results. This course, I maintain, will at least reward any one, amateur or otherwise. In the first place, a good and reliable microscope will be prerequisite. Secondly, a thorough practicable knowledge of the use of the instrument will be indispensible.

To commence with the gum, or soft pulp of a tooth. Formula No. 1. Procure a fresh pulp, sound and normal, and put this into alcohol (ordinary) and let it remain several days, when it becomes firm and tough. Then remove from the alcohol, and with a sharp rasor, slice into thin longitudinal sections, as thin as possible, observing the order in which they are cut. They are now ready for mounting and should be mounted in the order in which they are cut. Mount in balsam, label and number. This specimen will show sections of blood vessels, cellular tissue and portions of nerve filaments commencing with the first slice and follow the order of their mountings.

Formula No. 2. Procure a fresh pulp as before, and treat same way in alcohol, now remove and commence at one end and cut across at right angles to the pulp, and cut into thin slices laying them aside in the order in which they were cut. Then mount, label and number in the same order in which they were cut, as in the first process.

Formula No. 3. This time cut a pulp, prepared as above directed, obliquely from end to end, observing the same angle through the entire pulp, lay aside and mount as in the other cases.

In the above specimens the filamental septum may be imperfectly seen and studied, though not satisfactorily, as the cell contents will obscure the nerve fibrils though not entirely so. For the above processes, pulps of single teeth are preferable to begin with, then the molars may be studied in the same way. When the pulp is removed from the alcohol it is white and tough, and should be cut and mounted as soon as possible before it hardens too much.

Formula No. 4. Procure a fresh pulp and at once split it open from end to end, then lay it on a slip of glass slightly coated with balsam pitch and spread it out after slightly rounding the glass (something like a hunter stretches his coon skin on a barrel to dry) then remove as much of the cell contents as convenient by washing with a piece of sponge and acetic acid or ether, rubbing lengthwise with pulp. This will show more clearly the septum of filaments, and also show the countless openings through the pulp membrane where the fibrils pass out into the dentine. These specimens will not keep well for permanent use unless mounted in pitch on both sides, even then changes take place.

Formula No. 5. Fresh pulps may be dissected into small patches, or torn to pieces and mounted as above directed, which will show more or less of the structure of the interior of the pulp, patches of nerve fibres or filaments may be seen intact.

Formula No. 6. Remove fresh pulps and let them dry a day or so, then with a short pointed bistory or scissors split open the pulp from end to end, and spread out on coated glass as before directed, mount and label. In these specimens the openings through the membrane may be distinctly seen, also the system of nerves as heretofore described in former articles, though not entirely intact, but more or less distributed. The above are the leading features of preparing pulps for the microscope though many other methods may be employed, none of which will be entirely satisfactory to the beginner.

In the case of dryed pulps they may be put into water and swelled, then split open, dissected and examined.

A new set of formulas will now be given which will show the filamental system to perfection and *in situ*, and cell structures and blood vessels generally being obliterated by differentiation, only in some instances the vessels may be seen with their contents. As we lack terms to fully describe this process, I will suggest something new, as ossification and calcification neither are expressive. Dentification which is in use, and the most satisfactory, does not fully give the process; instead, I would suggest the terms *dentificatio pulpæ* or *dentum secundum*, meaning secondary dentine. Another form of hardened pulp, depending on another cause might be termed with propriety, *nodosa pulpa*, or granulated pulp, and may consist of one or more granules, round, or irregular in shape.

The former depending on wearing down of the tooth by mastication, and the latter depending on irritation from decay and other causes, is always confined to the limits of the pulp, and never adherent to the inner walls of the dentine. The former process may be considered as physiological and the latter as pathological. One anticipated by nature, or the *vis medicatrix naturæ*, and ample provision made when the process is normal. In the latter case, no such result is anticipated, and no constant saving provision instituted; on the contrary, is infrequent and accidental, happening, probably, in certain temperaments only.

In both cases the result is the same so far as these processes are viewed under the microscope, that is the filamental arrangement of nerves are in both cases intact, and show beautifully the plexuses of nerves occupying the interior of the pulp already described

The processes may be compared to petrifying processes in inorganic chemistry, soluble lime salts filling up the cells and around the nerve fibrils leaving these structures perfect and intact. Cell walls also remain, only their contents being replaced by lime, the blood vessels generally being filled with lime, or are absorbed and disappear.

The precise nature of these changes are not well understood, though the result may be readily comprehended. Without the aid of these secondary formations the true system of pulp structures never could be satisfactorily studied.

Specimens may be sawed with main-spring saws through crowns and roots when the pulp is ossified, including the pulp, showing the filamental system through the primary and secondary dentine in a perfect state of preservation. Section after section may be cut through the tooth and pulp in thin slices, and dressed down on a fine hone, first sawing through the centre, then saw off thin slabs on each side, dress down and saw as before—dress down again and saw until all the pulp is sawed up. These sections may be cemented to slips of glass with Canada balsam, pitch the dressed side to the glass, then dress down on a hone until light readily passes through it, then introduce under the instrument, and if not thin enough to show the nerves distinctly, dress down until they can be seen; if too thick, same as in preparing ordinary tooth specimens. This secondary nodes, or nodular dentine, is not true secondary dentine, though very similar, and might be included, perhaps, under the head of calcification, which may take place in any tissue, especially in advanced life, depending on similar causes that is stasis in the part, and deposit of lime, and is an accidental circumstance, and may serve as a protection to the pulp against further ravages of decay. In both forms of secondary dentine the remaining soft portions of the pulp retains its vitality to a certain extent, varying in different cases.

It will be found that in case of wearing of teeth of old persons there is evidence of tubular disturbance, especially near the former boundaries of pulp cavity, sometimes throughout the dentine. The connection and continuation of tubuli will be distinctly seen, and traced from primary to secondary dentine the regularity of the primary disappearing in the secondary dentine. Slips of glass, Canada balsam, spring wooden clamps, or clothes-pins, a spirit lamp, saw and hone is all that is necessary to prepare specimens. The pitch is formed by gently evaporating the balsam over a spirit lamp until when cold it becomes hard, and of a brown color.

